# Sensory impairment and risk of incident depression in older adults: a harmonized longitudinal analysis of 72,177 individuals across six international cohorts

**DOI:** 10.3389/fpubh.2026.1834248

**Published:** 2026-07-08

**Authors:** Lin Guo, Linlin Zhang, Zengying Zhuang, Yanjie Jiang, Linlin Yue

**Affiliations:** School of Management, Shandong Second Medical University, Weifang, Shandong, China

**Keywords:** dual sensory impairment, harmonized analysis, incident depression, sensory impairment, socioeconomic status

## Abstract

**Background:**

Evidence linking sensory impairment to depression is predominantly Western. We quantified longitudinal associations of single and dual sensory impairment (DSI) with incident depression using harmonized data from six countries.

**Methods:**

We analyzed 72,177 older adults (aged ≥50) from HRS (USA), ELSA (UK), SHARE (Europe), CHARLS (China), KLOSA (South Korea), and MHAS (Mexico). Participants free of depression at baseline (2010–2015) were followed for 2–10 years. Sensory status was categorized as no impairment, vision impairment only (VI), hearing impairment only (HI), and DSI. We used multivariable Cox models and individual participant data (IPD) meta-analysis to estimate pooled hazard ratios (HRs).

**Findings:**

Sensory impairment was consistently associated with an increased depression risk. DSI conferred the highest risk (pooled HR 1.39, 95% CI 1.32–1.46), followed by HI (HR 1.21, 1.16–1.27) and VI (HR 1.15, 1.11–1.20). Associations were stronger in Western cohorts (DSI HRs ≥1.84 in UK/USA) than in Asian and Latin American populations. Subgroup analyses showed higher DSI-associated risk among individuals with college education (HR 1.68) compared to those with lower education (HR 1.35).

**Interpretation:**

Sensory impairment, particularly DSI, is robustly associated with incident depression globally, though effects vary by geopolitical and socioeconomic context. Integrated sensory and mental health screening is critical for preventing late-life depression.

## 1 Introduction

Population aging represents one of the most profound demographic transitions of the 21st century. The World Health Organization (WHO) projects that between 2015 and 2050, the proportion of the world's population over 60 years will nearly double from 12 to 22%.^1^ Amidst this demographic shift, preserving mental well-being has emerged as a critical public health priority. Depression affects approximately 5.7% of older adults globally and stands as a leading contributor to the global burden of disease measured in Years Lived with Disability (YLDs).^2^ Concurrent with this mental health challenge is the escalating prevalence of sensory deficits. It is estimated that at least 2.2 billion people globally have a vision impairment,^3^ and over 1.5 billion live with some degree of hearing loss.^4^ Crucially, the WHO's *Integrated Care for Older People (ICOPE)* framework explicitly identifies sensory function as a foundational pillar of “intrinsic capacity,”^5^ suggesting that sensory health is not merely a physical concern but a modifiable leverage point for preventing broader psychological deterioration in later life.

While the nexus between sensory health and psychological wellbeing is increasingly recognized, current evidence remains fragmented. Recent longitudinal studies and meta-analyses have largely focused on single sensory modalities in isolation, establishing links between depression and specific vision-threatening conditions such as glaucoma and geographic atrophy (1–6), or detailing the psychological impact of hearing loss and chronic tinnitus (7–10). However, this “siloed” approach overlooks the synergistic burden of Dual Sensory Impairment (DSI), which may accelerate multimorbidity and cognitive decline more aggressively than single impairments (1–3, 11, 12). Furthermore, the majority of existing findings are derived from single-nation cohorts confined to specific cultural contexts—such as the UK Biobank (8, 12), CHARLS in China (6, 11, 13), or specific Korean populations (1–3). Although recent work has attempted to compare cumulative sensory burden between the US and China (14), there remains a paucity of harmonized, multi-continental studies that can systematically evaluate geopolitical heterogeneity. Additionally, the directionality of these associations is often clouded by reverse causality, as depressive symptoms themselves may precipitate sensory decline (1–3), necessitating rigorous longitudinal designs that exclude baseline depression to isolate incident risk.

To bridge these critical knowledge gaps, the present study capitalized on harmonized longitudinal data from six nationally representative cohorts spanning three continents: the Health and Retirement Study (HRS) in the USA, the English Longitudinal Study of Ageing (ELSA) in the UK, the Survey of Health, Ageing and Retirement in Europe (SHARE), the China Health and Retirement Longitudinal Study (CHARLS), the Korean Longitudinal Study of Ageing (KLOSA), and the Mexican Health and Aging Study (MHAS). By pooling a massive sample of 72,177 community-dwelling older adults and rigorously excluding those with depression at baseline, we established a robust framework to infer temporal directionality. Specifically, this study aimed to: (1) quantify the longitudinal associations between single (vision or hearing) and dual sensory impairments and the risk of incident depression; (2) systematicallly evaluate geopolitical heterogeneity to understand how these associations vary across distinct cultural and healthcare contexts; and (3) identify vulnerable subpopulations through stratified analyses of sociodemographic factors. This coordinated global analysis seeks to provide the most comprehensive evidence to date, moving beyond fragmented single-nation studies to inform integrated sensory and mental health policies for an aging world.

## 2 Methods

### 2.1 Data sources and study population

This study utilized harmonized longitudinal data from six nationally representative “sister studies” of aging, obtained from the Gateway to Global Aging Data platform (g2aging.org). These cohorts employed comparable sampling methodologies and survey instruments to facilitate cross-national research. The included datasets were: the Health and Retirement Study (HRS) in the United States; the English Longitudinal Study of Ageing (ELSA) in the United Kingdom; the Survey of Health, Ageing and Retirement in Europe (SHARE), covering multiple European countries; the China Health and Retirement Longitudinal Study (CHARLS) in China; the Korean Longitudinal Study of Ageing (KLOSA) in South Korea; and the Mexican Health and Aging Study (MHAS) in Mexico. To ensure strict comparability of measures across disparate cultural contexts, we utilized the Harmonized Data Files for each cohort, in which variables were retrospectively harmonized to align with RAND HRS definitions.

The baseline for the analysis was centered around the 2010–2012 period to maximize temporal synchronization: Wave 10 (2010) for HRS, Wave 5 (2010) for ELSA, Wave 4 (2011) for SHARE, Wave 1 (2011) for CHARLS, and Wave 3 (2010) for KLoSA. For MHAS, due to survey intervals, Wave 4 (2015) served as the baseline. Participants were followed up until the subsequent wave (approximately 2–4 years later) to ascertain incident depression. We applied strict exclusion criteria to the initial pooled sample: (1) age younger than 50 years; (2) presence of depressive symptoms at baseline (prevalent cases); and (3) missing data on sensory status, depression scores, or key covariates. The final analytical sample comprised 72,177 community-dwelling older adults.

#### 2.1.1 Ethical considerations

This study involved the secondary analysis of de-identified, publicly available data and was therefore considered exempt from further ethical review. No additional ethical approval was required for the present secondary analysis, as the study used existing anonymized data and did not involve new participant recruitment, direct contact, or intervention. The original data collection for each cohort was approved by their respective Institutional Review Boards (IRBs) or ethics committees: the University of Michigan (HRS), the London Multicentre Research Ethics Committee (ELSA), the Ethics Council of the Max Planck Society (SHARE), the Ethical Review Committee of Peking University (CHARLS), the Research Ethics Committee of the Korea Employment Information Service (KLOSA), and the University of Texas Medical Branch alongside the Instituto Nacional de Estadística y Geografía (MHAS). All participants provided written informed consent at the time of recruitment. All data were accessed and analyzed in accordance with the relevant cohort data-use agreements, access policies, and ethical requirements.

### 2.2 Sample selection and exclusion criteria

A rigorous, harmonized sample selection protocol was applied across all six cohorts. From the initial pooled dataset, we sequentially excluded observations with invalid follow-up durations (≤0 years) or those falling outside the designated baseline waves to ensure a valid longitudinal design. To ascertain incident depression, participants with prevalent depressive symptoms at baseline were strictly excluded. The sample was further restricted to community-dwelling adults aged 50 years or older. Finally, participants with missing data on sensory status, depression scores, or key covariates (including sociodemographics, health behaviors, and chronic conditions) were removed to ensure data completeness. A detailed step-by-step flowchart of the inclusion and exclusion process for each cohort is provided in Appendix B. The final analytical sample comprised 72,177 participants.

### 2.3 Measures

#### 2.3.1 Assessment of incident depression (outcome)

The primary outcome was incident depression, defined as the new onset of depressive symptoms during the follow-up period among participants who were free of depression at baseline. Depression was assessed using harmonized versions of validated symptom scales adapted for each cohort. For HRS, ELSA, and KLoSA, the Center for Epidemiologic Studies Depression Scale (CES-D) was employed (8-item version for HRS and ELSA; 10-item version for KLoSA and CHARLS; 9-item version for MHAS). For SHARE, the EURO-D scale was used. Consistent with established protocols in the Gateway to Global Aging Data, total scores were calculated for each participant, and validated cut-off points (CES-D 10 ≥10 for CHARLS and KLoSA; CES-D 8 ≥4 for HRS and ELSA; EURO-D ≥4 for SHARE; and CES-D 9 ≥5 for MHAS.) were applied to generate a binary variable (depressed vs. non-depressed). Participants with scores exceeding the threshold at any follow-up wave were classified as having incident depression. The date of onset was defined as the interview date of the wave in which the participant first screened positive.

#### 2.3.2 Assessment of sensory impairment (exposure)

The primary exposure was self-reported sensory impairment at baseline. Participants rated their eyesight and hearing (with corrective aids) on a Likert scale (e.g., excellent to poor). Consistent with established protocols in harmonized aging research, we dichotomized these responses. Generally, responses of “fair” or “poor” were classified as impaired. Notably, for the MHAS cohort, a stricter definition was necessitated by the data distribution characteristics, where impairment was defined based on reports of functional limitation or total sensory loss. Based on these assessments, a four-category variable was constructed: (1) No Sensory Impairment; (2) Vision Impairment Only (VI); (3) Hearing Impairment Only (HI); and (4) Dual Sensory Impairment (DSI). Although these harmonized self-reported measures improved cross-cohort comparability, they may not fully eliminate differences in reporting thresholds across cultural contexts. Therefore, the same response category may not necessarily correspond to an identical level of objectively measured sensory function across countries.

Sociodemographic factors included age (continuous years), sex (male/female), marital status (dichotomized into married/partnered vs. other [single, widowed, or divorced]), and educational attainment [standardized into three levels: less than high school, high school/vocational, and college or above based on the International Standard Classification of Education (ISCED)]. Household Wealth was calculated as the sum of all assets minus debts; to account for cross-national economic disparities, wealth was standardized by calculating within-country tertiles (low, middle, high).

Health and lifestyle factors included employment status (currently working vs. retired/not working), Smoking Status (current smoker vs. non-smoker), and chronic conditions, operationalized as a count of doctor-diagnosed chronic diseases (including hypertension, diabetes, cancer, lung disease, heart disease, stroke, and arthritis).

### 2.4 Statistical analysis

Statistical analyses were performed using Stata version 17.0 (StataCorp, TX, USA). Statistical significance was set at a two-sided *P* < 0.05. The analysis proceeded in five stages. Missing data were handled using complete case analysis. Survey weights were not applied in the pooled meta-analysis due to the lack of harmonized weighting variables across all cohorts, which is a common limitation in international IPD meta-analyses.

First, baseline characteristics of participants were summarized by cohort. Continuous variables were presented as means with standard deviations (SD), and categorical variables as frequencies with percentages.

Second, to examine the longitudinal association between sensory impairment and incident depression, we employed a two-stage random-effects individual participant data (IPD) meta-analysis. In the first stage, Cox proportional hazards regression models were fitted separately within each of the six cohorts to estimate cohort-specific hazard ratios (HRs) and 95% confidence intervals (CIs), allowing each estimate to reflect the cohort's own follow-up structure. In the second stage, these cohort-specific estimates were pooled using a random-effects meta-analysis, which accounted for between-cohort heterogeneity, including differences in depression assessment tools and follow-up intervals.

Third, in the second stage, cohort-specific estimates were pooled using a random-effects meta-analysis (DerSimonian-Laird method) to generate global summary estimates for Vision Impairment Only, Hearing Impairment Only, and Dual Sensory Impairment. Heterogeneity across cohorts was quantified using the *I*^2^ statistic and Cochran's *Q* test.

Fourth, to visualize the temporal accumulation of risk, Kaplan–Meier failure curves were plotted for each sensory status group, illustrating the cumulative incidence of depression over the follow-up period.

Fifth, we conducted subgroup analyses on the pooled dataset (*N* = 72,177) to explore potential effect modifiers. Stratified Cox models were fitted by age (< 65 vs. ≥65 years), sex, marital status, education, employment, smoking, and wealth tertiles, adjusting for country to account for clustering. The significance of differences between subgroups was assessed using *P*-values for interaction.

Finally, to ensure the robustness of our findings, we performed two sensitivity analyses: (1) a lag analysis excluding participants who developed depression or were censored within the first 2 years to minimize reverse causality; and (2) multivariable logistic regression models to estimate odds ratios (ORs), thereby verifying associations independent of survival time assumptions.

## 3 Results

### 3.1 Baseline characteristics of the study population

The final analytical sample comprised 72,177 community-dwelling older adults across six harmonized cohorts. Baseline characteristics exhibited substantial heterogeneity across geopolitical regions (Table 1). Participants in the Chinese cohort (CHARLS) were the youngest (mean age: 61.51 years), whereas those in the UK (ELSA) and USA (HRS) were older (mean age: 66.42 and 66.06 years, respectively). Socioeconomic disparities were pronounced; educational attainment followed a clear gradient, with the vast majority of participants in China (86.00%) and Mexico (83.55%) having less than a high school education, compared to only 16.40% in the USA.

**Table 1:** Baseline characteristics of the study population stratified by cohort (*N* = 72,177).

Variables	CHARLS (*N* = 6,055)	HRS (*N* = 15,617)	ELSA (*N* = 6,910)	SHARE (*N* = 31,243)	KLOSA (*N* = 4,856)	MHAS (*N* = 7,496)
Age (years)	61.51 ± 8.03	66.06 ± 10.74	66.42 ± 9.02	65.13 ± 9.30	63.98 ± 9.62	65.77 ± 8.97
Chronic conditions count	0.84 ± 0.94	1.73 ± 1.31	1.06 ± 1.01	1.04 ± 1.06	0.84 ± 0.95	1.23 ± 1.08
Sensory status						
No impairment (ref)	1,161(19.17)	10,886(69.17)	5,153(74.57)	19,372(62.00)	2,117(43.60)	6,786(90.53)
Vision impairment only	1,855(30.64)	1,990(12.74)	450(6.51)	6,539(20.93)	1,280(26.36)	311(4.15)
Hearing impairment only	366(6.04)	1,702(10.90)	1,042(15.08)	3,037(9.72)	1,308(26.94)	379(5.06)
Dual sensory impairment	2,673(44.15)	1,039(6.65)	265(3.84)	2,295(7.35)	151(3.11)	20(0.27)
Employment status						
Yes	3,738(61.73)	6,890(44.12)	2,578(37.31)	11,173(35.76)	2,390(49.22)	3,224(43.01)
No	2,317(38.27)	8,727(55.88)	4,332(62.69)	20,070(64.24)	2,466(50.78)	4,272(56.99)
Smoking status						
Current smoker	2,026(33.46)	2,139(13.70)	790(11.43)	5734(18.35)	890(18.33)	904(12.06)
Non-smoker	4,029(66.54)	13,478(86.30)	6,120(88.57)	25509(81.65)	3,966(81.67)	6,592(87.94)
Sex						
Male	3,344(55.23)	6,750(43.22)	3,278(47.44)	15,075(48.25)	2,256(46.46)	3,497(46.65)
Female	2,711(44.77)	8,867(56.78)	3,632(52.56)	16,168(51.75)	2,600(53.54)	3,999(53.35)
Marital status						
Married/partnered	5,399(89.17)	9,683(62.00)	4,871(70.49)	22,704(72.67)	3,987(82.10)	5,297(70.66)
Other	656(10.83)	5,934(38.00)	2,039(29.51)	8,539(27.33)	869(17.90)	2,199(29.34)
Household wealth (tertiles)						
Low (tertile 1)	2,020(33.36)	5,206(33.34)	2,304(33.34)	10,415(33.34)	1,657(34.12)	2,500(33.35)
Middle (tertile 2)	2,018(33.33)	5,207(33.34)	2,303(33.33)	10,414(33.33)	1,590(32.74)	2,499(33.34)
High (tertile 3)	2,017(33.31)	5,204(33.32)	2,303(33.33)	10,414(33.33)	1,609(33.13)	2,497(33.31)
Educational attainment						
Less than high school	5207(86.00)	2,561(16.40)	2,001(28.96)	11,759(37.64)	2,807(57.80)	6,263(83.55)
High school/vocational	708(11.69)	9,236(59.14)	3,539(51.22)	12,430(39.78)	1,514(31.18)	317(4.23)
College or above (high)	140(2.31)	3,820(24.46)	1,370(19.83)	7,054(22.58)	535(11.02)	916(12.22)
Incident depression						
Yes	2805(46.33)	3,319(21.25)	1,154(16.70)	9,687(31.01)	2,096(43.16)	1,345(17.94)
No	3250(53.67)	12,298(78.75)	5,756(83.30)	21,556(68.99)	2,760(56.84)	6,151(82.06)

Data are presented as mean (standard deviation) for continuous variables and frequency (column percentage) for categorical variables. CHARLS, China Health and Retirement Longitudinal Study; HRS, Health and Retirement Study; ELSA, English Longitudinal Study of Ageing; SHARE, Survey of Health, Ageing and Retirement in Europe; KLoSA, Korean Longitudinal Study of Ageing; MHAS, Mexican Health and Aging Study. Household Wealth represents within-country tertiles; for CHARLS, per capita household consumption was used as a proxy for economic status. Chronic conditions count represents the number of doctor-diagnosed chronic diseases. Incident depression refers to new-onset depressive symptoms during the follow-up period.

Health profiles also varied distinctively. The burden of chronic conditions was highest in the HRS (mean count: 1.73) and lowest in CHARLS and KLoSA (mean count: 0.84). Regarding sensory status, the prevalence of dual sensory impairment (DSI) showed striking regional differences, being highest in CHARLS (44.15%), followed by SHARE (7.35%) and HRS (6.65%), and lowest in MHAS (0.27%). The cumulative incidence of depression during follow-up also ranged widely, from 16.70% in ELSA to 46.33% in CHARLS and 43.16% in KLoSA. These variations reflect the inherent cross-national diversity in demographic structures, health reporting behaviors, and socioeconomic contexts.

### 3.2 Correlations and multicollinearity assessment

**Figure 1:**
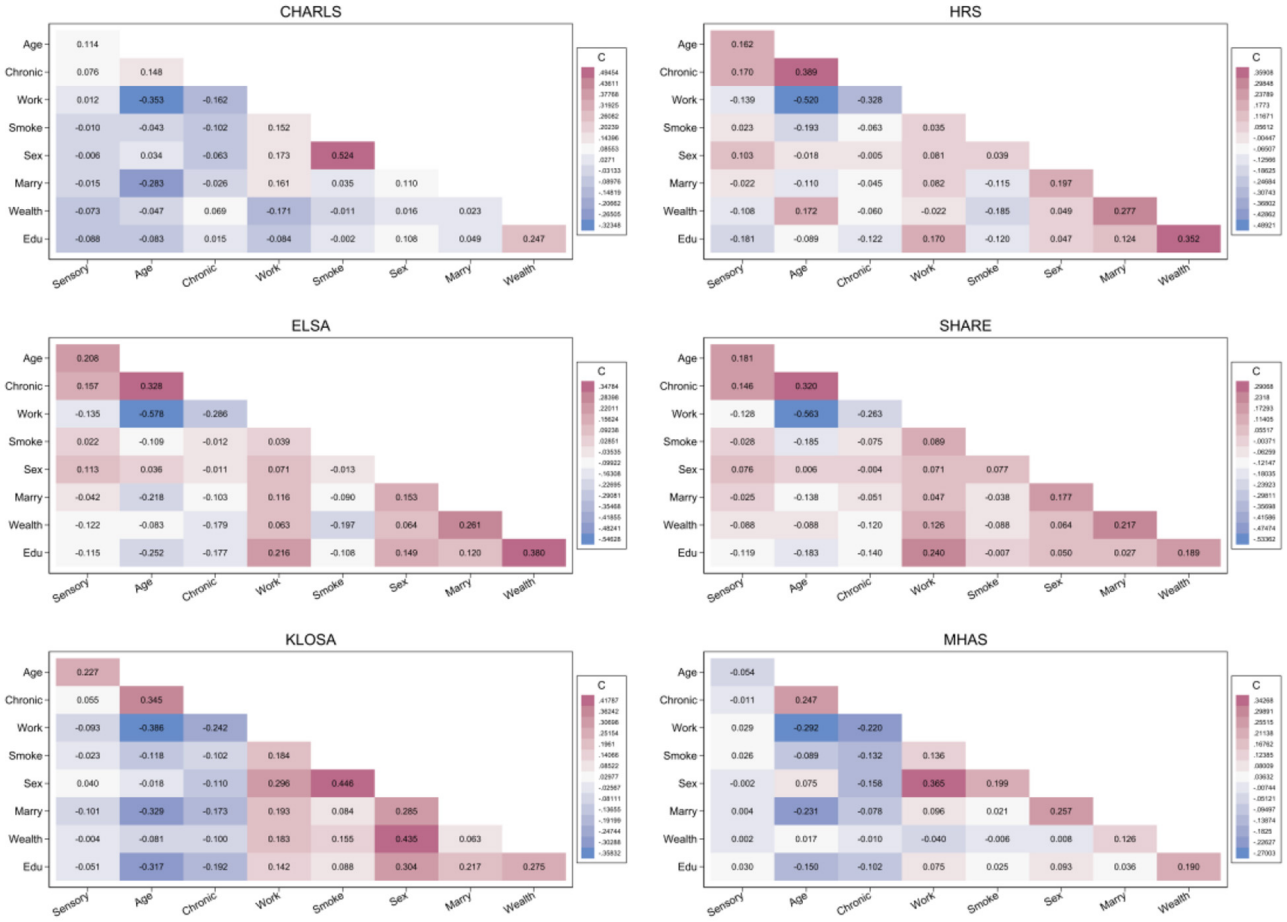
Heatmaps of pairwise Pearson correlation coefficients between study variables across six international cohorts. The figure displays the correlation matrices for CHARLS, HRS, ELSA, SHARE, KLoSA, and MHAS. The color gradient represents the strength and direction of the correlation: red indicates a positive correlation, while blue indicates a negative correlation. The numerical values within each cell represent the Pearson correlation coefficients. Sensory, Sensory Status (0 = no impairment, 1 = vision only, 2 = hearing only, 3 = dual impairment); age, age (years); chronic, number of chronic conditions; work, employment status (yes/no); smoke, smoking status (current smoker/non-smoker); sex, sex (male/female); marry, marital status (married or partnered/other); wealth, household wealth (tertiles); edu, educational attainment (low/middle/high).

To evaluate the independence of covariates and rule out potential multicollinearity, we examined pairwise Pearson correlation coefficients among the key study variables across all six cohorts (Figure 1). The correlation heatmaps generally displayed weak-to-moderate associations between variables. The strongest correlations were observed between sociodemographic factors, such as Age and Work (Employment Status), which showed expected negative correlations (ranging from −0.13 to −0.35), and between sex and smoke (smoking status) in specific cohorts (e.g., *r* = 0.45 in KLOSA). Importantly, the key exposure variable, sensory (sensory status), demonstrated low correlations with all other covariates (|*r*| < 0.20 in most cases), suggesting that sensory impairment is distinct from potential confounders. Across all cohorts, no pairwise correlation coefficient exceeded the threshold of 0.70. Consequently, variance inflation factors (VIFs) were presumed to be within acceptable limits, indicating that multicollinearity was not a concern for the subsequent multivariable Cox regression models.

### 3.3 Pooled associations stratified by sensory impairment type

**Figure 2:**
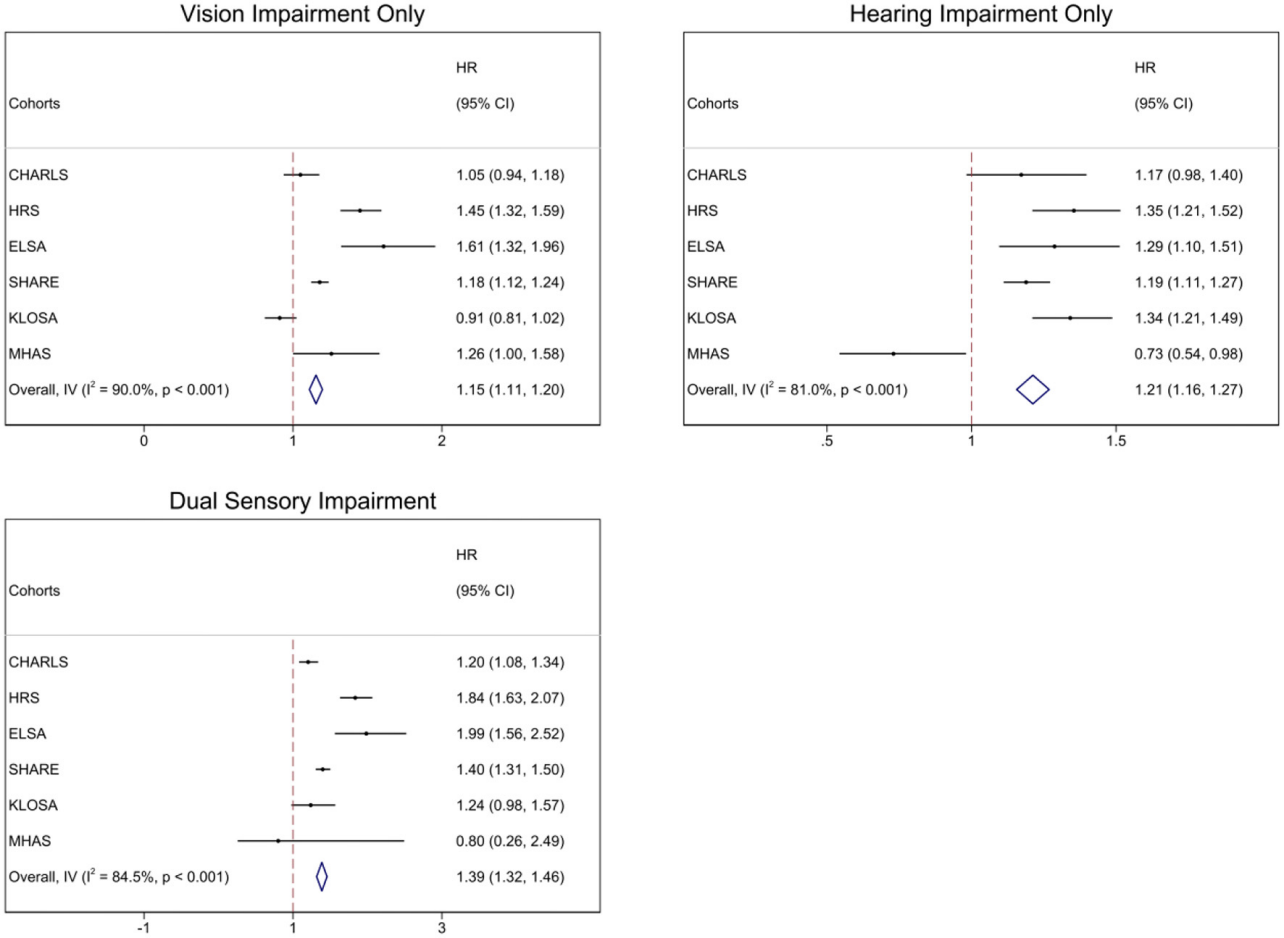
Forest plots of hazard ratios for incident depression associated with single and dual sensory impairments across six cohorts. The plots display cohort-specific and pooled hazard ratios (HRs) with 95% confidence intervals (CIs) for vision impairment only **(top left)**, hearing impairment only **(top right)**, and dual sensory impairment **(bottom left)**, compared with no sensory impairment. The diamond represents the overall pooled estimate derived from random-effects meta-analysis. *I*^2^ statistics indicate the degree of heterogeneity across cohorts. The analysis included a total of 72,177 participants. HR, hazard ratio; CI, confidence interval; CHARLS, China Health and Retirement Longitudinal Study; HRS, Health and Retirement Study; ELSA, English Longitudinal Study of Ageing; SHARE, Survey of Health, Ageing and Retirement in Europe; KLoSA, Korean Longitudinal Study of Ageing; MHAS, Mexican Health and Aging Study.

Figure 2 presents the cohort-specific and pooled hazard ratios (HRs) for incident depression associated with single and dual sensory impairments across the six cohorts (*N* = 72,177). The random-effects meta-analysis identified dual sensory impairment (DSI) as having the strongest association with of incident depression, with a pooled HR of 1.39 (95% CI: 1.32–1.46). Significant pooled associations were also observed for hearing impairment only (HR = 1.21, 95% CI: 1.16–1.27) and vision impairment only (HR = 1.15, 95% CI: 1.11–1.20).

Substantial heterogeneity was detected across all impairment categories (*I*^2^ > 80%, *p* < 0.001), with notable variations between cohorts. For Vision Impairment Only, the associations were robust in Western populations, led by the UK (ELSA: HR = 1.61, 95% CI: 1.32–1.96) and the USA (HRS: HR = 1.45, 95% CI: 1.32–1.59). In contrast, the association was not statistically significant in the Korean cohort (KLoSA: HR = 0.91, 95% CI: 0.81–1.02) or the Chinese cohort (CHARLS: HR = 1.05, 95% CI: 0.94–1.18).

Regarding Hearing Impairment Only, elevated risks were consistently observed in Western cohorts and Korea (HRs ranging from 1.19 to 1.35). However, the Mexican cohort (MHAS) exhibited a divergent pattern, showing a protective point estimate (HR = 0.73, 95% CI: 0.54–0.98). For DSI, the magnitude of risk was highest in the UK and USA (HRs ≥1.84), intermediate in Europe (HR = 1.40), and lowest in China (HR = 1.20) and Mexico (HR = 0.80, 95% CI: 0.26–2.49).

### 3.4 Cumulative incidence of depression stratified by sensory status

**Figure 3:**
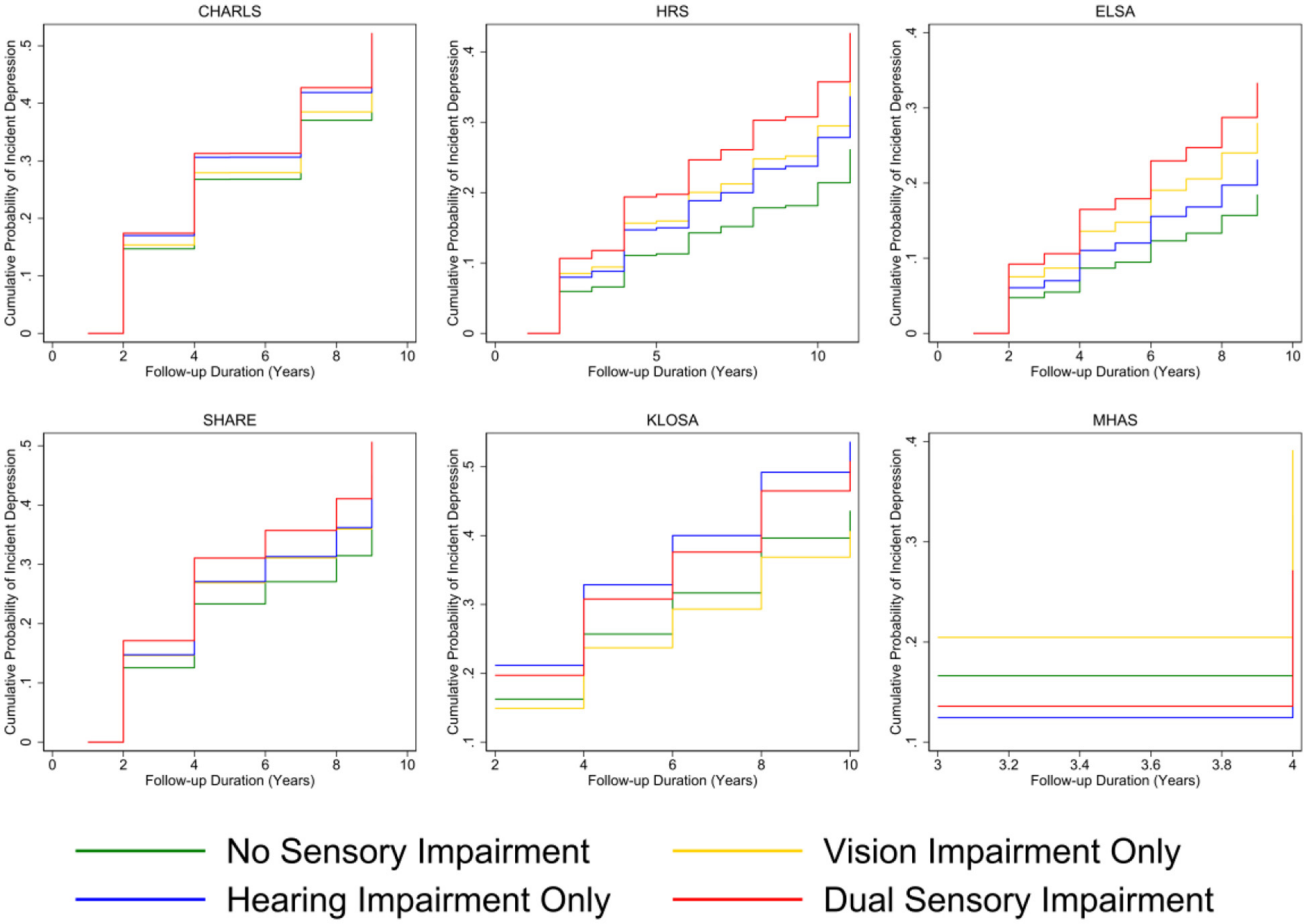
Cumulative incidence of incident depression stratified by baseline sensory status across six international cohorts. Kaplan–Meier failure curves display the cumulative probability of developing depression over the follow-up duration (years) for each cohort. Participants are stratified into four mutually exclusive groups: no sensory impairment (green line), vision impairment only (yellow line), hearing impairment only (blue line), and dual sensory impairment (red line). The distinct separation of curves highlights the differential rates of depression onset, with steeper slopes indicating higher risk accumulation. CHARLS, China Health and Retirement Longitudinal Study; HRS, Health and Retirement Study; ELSA, English Longitudinal Study of Ageing; SHARE, Survey of Health, Ageing and Retirement in Europe; KLoSA, Korean Longitudinal Study of Ageing; MHAS, Mexican Health and Aging Study.

Kaplan-Meier estimates revealed distinct longitudinal trajectories of depression risk accumulation across the six cohorts (Figure 3). In the Western cohorts—specifically HRS (USA), ELSA (UK), and SHARE (Europe)—a clear and consistent risk hierarchy was observed. Participants with dual sensory impairment (DSI; red line) exhibited the steepest cumulative incidence of depression over the follow-up period, followed by those with single impairments (yellow/blue lines), while those with No Sensory Impairment (green line) maintained the lowest risk profile. This stepwise separation suggests a “dose-response” relationship, where the accumulation of sensory deficits progressively accelerates the onset of depression.

In contrast, distinct regional patterns emerged in Asian and Latin American populations. The KLOSA (Korea) cohort displayed a hearing-dominant risk profile, where the trajectory for hearing impairment only (blue line) closely tracked with DSI, both significantly surpassing the risk for vision impairment or no impairment. In CHARLS (China), while DSI participants showed elevated risk, the confidence bands between impairment groups appeared more compressed compared to Western cohorts, indicating a less differentiated risk accumulation. Notably, the MHAS (Mexico) cohort presented a divergent pattern driven by Vision Impairment Only (yellow line), which showed the highest cumulative probability, whereas the trajectory for DSI was unstable, likely reflecting the low prevalence of dual impairment in this specific sample.

### 3.5 Subgroup analyses

**Figure 4:**
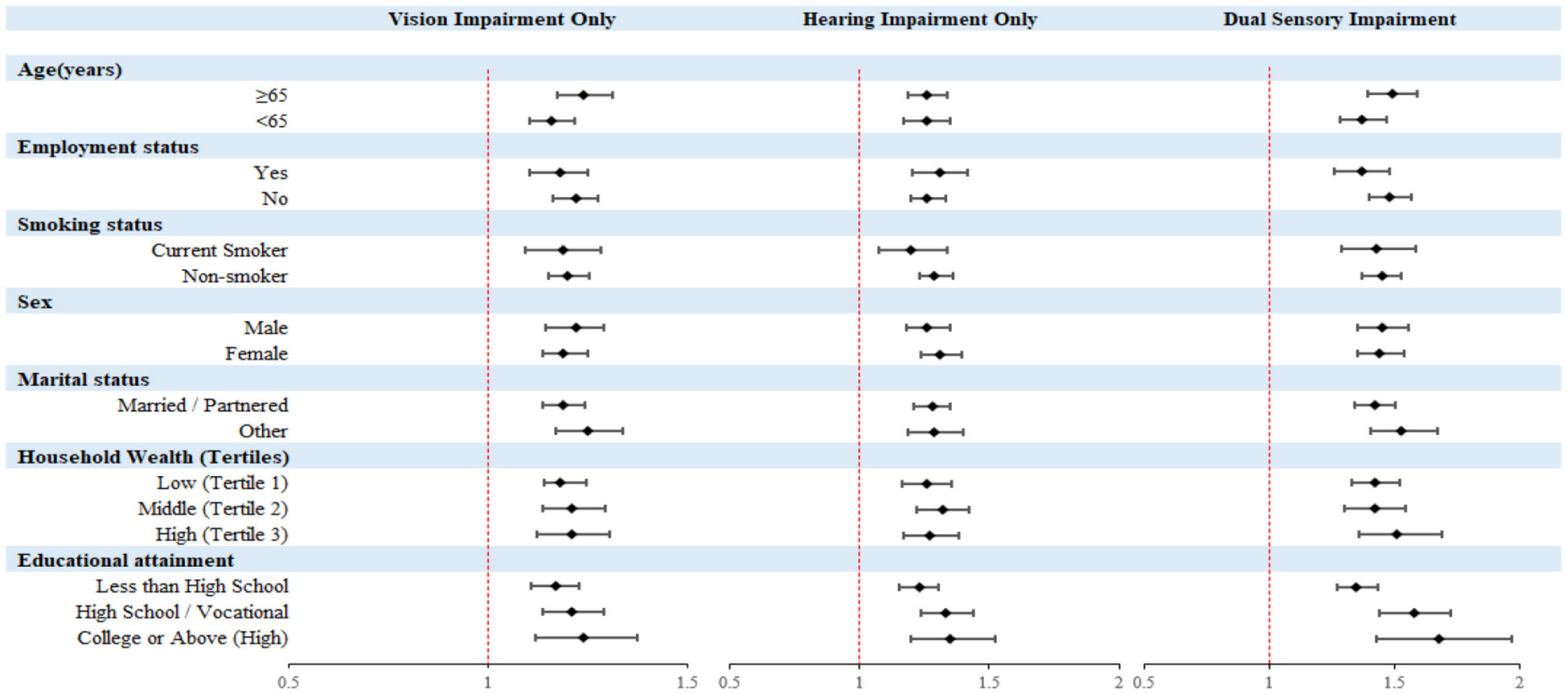
Forest plot illustrating the risk of incident depression associated with dual sensory impairment (DSI) stratified by key sociodemographic characteristics. The figure displays the hazard ratios (HRs, represented by squares) and 95% confidence intervals (CIs, represented by horizontal lines) for the association between dual sensory impairment (vs. no impairment) and incident depression across various subgroups. The vertical dashed line at HR, 1.0 indicates the null value; estimates entirely to the right of this line indicate a statistically significant increase in risk. Models were based on the pooled dataset (*N* = 72,177) and adjusted for age, sex, marital status, education, wealth, employment, smoking, chronic conditions, and country, excluding the stratification variable in each respective analysis. The size of the square is proportional to the weight of each subgroup in the analysis. CI, confidence interval; HR, hazard ratio.

To evaluate the robustness of the associations and identify potential vulnerable populations, we performed subgroup analyses stratified by age, sex, marital status, socioeconomic factors (education, wealth, employment), and smoking status on the pooled dataset (Figure 4 and Appendix C).

#### 3.5.1 Consistency of associations

The positive association between sensory impairment and incident depression was remarkably consistent across all stratified subgroups. In every subgroup, dual sensory impairment (DSI) consistently conferred the highest risk of depression, followed by hearing impairment only (HI) and vision impairment only (VI). This confirms that sensory impairment is a universal risk factor for depression, independent of sociodemographic backgrounds.

#### 3.5.2 Effect modification by sociodemographic factors

Although the direction of the association remained stable, the magnitude of the risk varied notably across certain subgroups:

Age: the impact of dual sensory impairment was more pronounced among older adults (≥65 years: HR 1.49, 95% CI 1.39–1.59) compared to their younger counterparts (< 65 years: HR 1.37, 95% CI 1.28–1.47).

Socioeconomic status (education gradient): interestingly, we observed a “risk gradient” related to educational attainment. The relative risk of depression associated with DSI appeared to increase with education level, being highest among those with college education or above (HR 1.68, 95% CI 1.43–1.97) and lowest among those with less than high school education (HR 1.35, 95% CI 1.27–1.43). A similar but less pronounced pattern was observed for household wealth (high wealth HR 1.51 vs. low wealth HR 1.42).

Social support (marital status): participants who were single, widowed, or divorced (“other”) exhibited a higher risk of depression from DSI (HR 1.53) compared to those who were married or partnered (HR 1.42), suggesting that spousal support may buffer some of the psychological impact of sensory loss.

Employment: the risk associated with DSI was also elevated among those not currently employed (HR 1.48) compared to the employed group (HR 1.37).

#### 3.5.3 Sex differences

Associations for dual sensory impairment were similar between sexes (male HR 1.45 vs. female HR 1.44). However, for single sensory impairments, men appeared slightly more sensitive to vision loss (male HR 1.22 vs. female HR 1.19), while women showed a marginally higher risk estimate for hearing loss (female HR 1.31 vs. male HR 1.26). These patterns may reflect social and functional roles: men often link vision to independence and daily functioning, making visual loss more psychologically impactful, whereas women frequently rely on verbal communication for social connectedness, making hearing loss a greater barrier to engagement.

### 3.6 Sensitivity analysis

#### 3.6.1 Exclusion of early events

To mitigate the potential bias of reverse causality (protopathic bias), we conducted a landmark sensitivity analysis by excluding participants who developed depression or were censored within the first 2 years of follow-up (Appendix D). The pooled results remained robust and statistically significant, closely mirroring the primary findings. Dual sensory impairment continued to exhibit the strongest association with incident depression, with a pooled hazard ratio of 1.34 (95% CI: 1.25–1.43). Significant associations also persisted for hearing impairment only (pooled HR = 1.14, 95% CI: 1.07–1.21) and vision impairment only (pooled HR = 1.13, 95% CI: 1.08–1.19). Although the magnitude of these estimates was slightly attenuated compared to the full sample analysis, the consistency of the direction and significance suggests that the observed associations are unlikely to be driven by reverse causation.

#### 3.6.2 Logistic regression models

To verify the robustness of our findings independent of the proportional hazards assumption inherent in Cox regression, we performed a sensitivity analysis using multivariable logistic regression models (Appendix E). The results corroborated the primary analysis, yielding consistent associations with slightly larger effect magnitudes, as expected with Odds Ratios (ORs) in a common outcome setting. Dual Sensory Impairment remained the strongest predictor of incident depression, with a pooled OR of 1.56 (95% CI: 1.45–1.66). Similarly, robust pooled associations were observed for Hearing Impairment Only (OR = 1.24, 95% CI: 1.17–1.32) and Vision Impairment Only (OR = 1.19, 95% CI: 1.13–1.25). The cohort-specific patterns, including the high risks in Western populations and the divergent patterns in Asian and Latin American cohorts, persisted in these logistic models, confirming the stability of the observed geopolitical gradients.

## 4 Discussion

### 4.1 Principal findings

To the best of our knowledge, this is the first study to harmonize data from six nationally representative cohorts across three continents to systematically quantify the longitudinal association between sensory impairment and incident depression. In this pooled analysis of 72,177 community-dwelling older adults, we found robust evidence that sensory impairment shows an independent and universal longitudinal association with incident depression, persisting after rigorous adjustment for sociodemographic factors, health behaviors, and chronic conditions. Our results revealed a clear “dose-response” risk hierarchy: dual sensory impairment (DSI) conferred the highest risk (pooled HR = 1.39), followed by hearing impairment only (HI, HR = 1.21) and vision impairment only (VI, HR = 1.15). While the direction of this association was largely consistent globally, we observed notable geopolitical heterogeneity, with risks being more pronounced in Western cohorts compared to Asian and Latin American populations. Furthermore, our subgroup analyses uncovered a counterintuitive “risk gradient” where the adverse psychological impact of dual sensory impairment was significantly amplified among individuals with higher educational attainment, suggesting complex interactions between sensory function, socioeconomic status, and psychological resilience.

### 4.2 Comparison with previous studies

Single sensory impairment: our findings regarding the independent contributions of single sensory modalities align with and extend recent evidence from specific national contexts. Regarding vision impairment, our observation of a 15% increased risk of depression corroborates the findings of Hashemi et al. (5), who reported that older adults with VI in Iran exhibited significantly higher scores for anxiety and depression, independent of age and sex. Similarly, in a nationwide Korean cohort, Wang et al. (1–3) demonstrated that patients with glaucoma and concurrent VI had an adjusted HR of 1.164 for depression, with risk escalating alongside VI severity. Zhao et al. (6) further elucidated the mechanism in the Chinese population using CHARLS data, identifying that the VI-depression association is partially mediated by limitations in social engagement and physical activity. Our study generalizes these country-specific associations to a global scale, confirming that vision loss fundamentally compromises mental wellbeing across diverse cultural settings.

Regarding hearing impairment, our pooled HR of 1.21 is consistent with the meta-analysis by Li et al. (7), which concluded that age-related hearing loss significantly increases depressive symptom scores (Hedges' *g* = 0.52), particularly in women. Recent cohort studies reinforce this link; for instance, Lu et al. (15) reported a 1.5-fold increase in depression odds among Chinese older adults with hearing loss, while Chen et al. (8) observed a similar hazard ratio (HR = 1.14) in the UK Biobank, noting that hearing loss was also associated with altered brain morphology. Furthermore, Kim and Hwang (10) emphasized that digital device use did not buffer this negative impact, suggesting that the psychological burden of hearing loss is difficult to mitigate through technology alone. Our results confirm that HI remains a robust risk factor for depression even after adjusting for a wider range of chronic conditions and socioeconomic variables across six distinct healthcare systems.

The synergistic burden of dual sensory impairment: a critical finding of our study is that the co-occurrence of vision and hearing loss (DSI) is synergistically associated with depression risk. This supports the concept of “cumulative sensory load.” Our results resonate with the recent work of Zhou et al. (14), who analyzed longitudinal data from HRS and CHARLS and found that cumulative average sensory impairments were associated with a 39% increase in incident depressive symptoms in China and 10% in the US. Similarly, Wang et al. (1–3) utilized CHARLS data to show that early DSI significantly raises the risk of multimorbidity, suggesting a systemic decline in health reserves. The psychological pathway of this synergistic effect is further supported by Mao et al. (11), whose moderated chain-mediated model demonstrated that anxiety and depression jointly mediate the relationship between DSI and cognitive decline. Moreover, Du et al. (16) highlighted the economic consequences, finding that depression mediates the association between DSI and catastrophic healthcare expenditures. Collectively, these studies, alongside our survival analysis, suggest that DSI represents a state of severe functional deprivation that accelerates both psychological deterioration and broader health decline more aggressively than single impairments.

### 4.3 Interpretation of geopolitical heterogeneity and subgroup findings

#### 4.3.1 Understanding the geopolitical gradient

While sensory impairment was universally associated with elevated depression risk, the magnitude of this association exhibited a distinct geopolitical gradient, with stronger effect sizes observed in Western cohorts (HRS, ELSA) compared to Asian (CHARLS) and Latin American (MHAS) populations. This heterogeneity may largely stem from cultural differences in self-reporting standards and the resulting variations in baseline prevalence. In our study, applying the “fair or poor” criterion resulted in a remarkably high prevalence of dual sensory impairment (DSI) in CHARLS (44.15%), which aligns with the 63.59% DSI prevalence reported by He et al. (13) using similar definitions in the same cohort. In contrast, the prevalence in Western cohorts was much lower (< 10%). The inclusion of a substantial proportion of participants reporting “Fair” sensory function in the CHARLS impairment group likely diluted the clinical severity of the exposure. This “phenotypic dilution” may explain why the hazard ratio in China (HR = 1.20) was more modest compared to the stricter, clinically severe impairment definitions captured in the US and UK cohorts (HRs ≥ 1.84). Furthermore, Zhou et al. (14) recently highlighted that while cumulative sensory impairment predicts depressive symptoms in both the US and China, the trajectories differ, suggesting that distinct environmental stressors and healthcare access in developing nations might interact differently with sensory decline. The MHAS cohort demonstrated divergent associations, including a potentially protective association for hearing impairment, which may be attributable to the relatively low prevalence of dual sensory impairment, limited statistical power, survivor bias, or cohort-specific reporting patterns. Nevertheless, despite variability in effect magnitude, the overall direction of association between sensory impairment and incident depression remained broadly consistent across most cohorts, supporting the robustness of the main findings.

#### 4.3.2 The “paradox” of socioeconomic status: why are the advantaged more vulnerable?

A striking and counterintuitive finding of our subgroup analysis was the “risk gradient” associated with socioeconomic status (SES): individuals with higher educational attainment (college or above: HR = 1.68) and greater household wealth faced a significantly higher relative risk of depression following DSI compared to their lower-SES counterparts. This observed trend may be partially explained through the lens of “status inconsistency” and the “loss of valued activities.” High-SES individuals often engage in cognitively demanding and socially active lifestyles that rely heavily on intact sensory function. Zhao et al. (6) demonstrated in a longitudinal mediation analysis that the association between vision impairment and depression in older adults is substantially mediated by limitations in social engagement (6.3%) and physical activity (21.3%). For highly educated individuals, sensory loss may impose a more profound disruption to these valued social and intellectual activities, leading to a steeper psychological decline. Moreover, Mao et al. (11) identified that DSI directly impacts cognitive ability, with anxiety and depression acting as key chain mediators. Since cognitive reserve is a core asset for high-SES individuals, the threat DSI poses to cognitive maintenance—as evidenced by the link between sensory deprivation and brain atrophy or functional connectivity loss described by Guo et al. (12) —may trigger greater psychological distress. Additionally, Du et al. (16) found that depression mediates the relationship between DSI and catastrophic healthcare expenditures; while high-SES individuals are financially more resilient, their higher expectations for health-related quality of life may render them more sensitive to the “health shock” of irreversible sensory failure.

### 4.4 Underlying mechanisms

The association between sensory impairment and depression is likely underpinned by a complex interplay of biopsychosocial mechanisms. Biologically, the “sensory deprivation hypothesis” suggests that prolonged lack of sensory input may induce structural and functional changes in the brain. Chen et al. (8) provided neuroimaging evidence from the UK Biobank, showing that hearing loss is associated with altered brain volume in regions processing emotion and auditory information, which directly correlates with anxiety and depression scores. This neurobiological pathway is further complicated by cognitive load. Mao et al. (11) elucidated a “moderated chain-mediated” pathway where dual sensory impairment precipitates cognitive decline, which in turn exacerbates anxiety and depression, creating a vicious cycle of mental deterioration. Similarly, Park et al. (17) highlighted that deficits in specific visual functions, such as stereoscopic vision, are significantly linked to cognitive dysfunction, further eroding the psychological resilience of older adults.

Psychosocially, the withdrawal from social participation is a dominant mediator. Lu et al. (15) emphasized that engagement in outdoor activities significantly buffers the depression risk associated with hearing loss; however, sensory impairment often forces individuals to retreat from such interactions due to communication embarrassment. Specific auditory symptoms also play a role; Cui and Du (9) identified chronic tinnitus as a critical mediator, noting that the persistent distress from phantom sounds significantly amplifies depression in those with hearing loss. Furthermore, the impact of these mechanisms may vary by gender. Hald et al. (18) observed that the link between sensory impairment and wellbeing is nuanced by gender roles, suggesting that men and women may internalize sensory loss differently within their relationship dynamics. Finally, Wang et al. (1–3) proposed that early sensory impairment serves as a sentinel for systemic multimorbidity, implying that the observed depression may also stem from the cumulative burden of deteriorating general health.

### 4.5 Implications for public health and clinical practice

Our findings have urgent implications for integrated geriatric care. Given the high prevalence of depression among those with specific ocular conditions—such as the increased risk observed in patients with glaucoma (1–3) and geographic atrophy (4)—ophthalmology and audiology clinics should implement routine mental health screenings. The “dose-response” relationship we identified suggests that interventions targeting even single impairments could prevent the escalation to dual impairment and severe depression. Technological interventions offer a promising avenue. Kim and Hwang (10) demonstrated that the use of digital devices can moderate the relationship between hearing loss and depression, suggesting that digital inclusion initiatives could serve as a compensatory mechanism for social connectedness. However, as noted by Scoresby et al. (19), in contexts like the COVID-19 pandemic, communication barriers (e.g., masking) can exacerbate anxiety, highlighting the need for adaptive communication strategies. From a policy perspective, Du et al. (16) revealed that depression mediates the link between dual sensory loss and catastrophic healthcare expenditure. Therefore, investing in early sensory rehabilitation (e.g., hearing aids, cataract surgery) is not only a clinical imperative but also a cost-effective strategy to mitigate the economic burden of mental health comorbidity. These findings also align closely with the WHO Integrated Care for Older People (ICOPE) framework. Sensory impairment should be regarded not only as a functional deficit, but also as a potential early marker of vulnerability to depression in later life. In primary care and community-based geriatric services, integrated screening pathways may therefore be warranted, whereby older adults reporting vision or hearing difficulties are also screened for depressive symptoms, and those presenting with depression are evaluated for potentially modifiable sensory deficits. This approach may be particularly important for individuals with dual sensory impairment, who may benefit from prioritization for multidomain assessment and early intervention.

### 4.6 Strengths and limitations

The major strengths of this study include the harmonization of data from six large-scale national cohorts, the rigorous exclusion of baseline depression to minimize reverse causality, and the comprehensive adjustment for confounders. However, several limitations must be acknowledged.

First, sensory status was self-reported, which may introduce measurement bias compared to audiometric or objective visual assessments. This issue is particularly prominent in cross-national research. Cultural norms, societal perceptions of aging, levels of health literacy, accessibility to medical diagnosis and assistive devices, as well as response styles, can all influence older adults' self-rated vision and hearing status. Nevertheless, self-reported metrics reflect the lived experience of functional difficulty, which is highly relevant to psychological wellbeing. Future studies should incorporate objective ophthalmological and audiometric assessments alongside culturally calibrated self-report scales to validate and further extend the findings of this study.

Second, the assessment of depression relied on different validated scales across cohorts, which may capture varying dimensions of the disorder. The CES-D, utilized in most cohorts, is known for its robust capture of somatic symptoms, such as sleep disturbances and fatigue. This may be particularly relevant for the Asian cohorts (CHARLS and KLoSA), where individuals often manifest psychological distress through physical complaints. Conversely, the EURO-D scale used in the European cohort (SHARE) emphasizes affective symptoms like guilt and tearfulness. While this heterogeneity in measurement could influence cross-national comparability, our use of harmonized thresholds and the consistent direction of Hazard Ratios across most cohorts suggest that the sensory-depression link transcends the specific instrument used for assessment.

Third, while we utilized lag analysis to address reverse causality, the bidirectional nature of this relationship cannot be fully ruled out. Wang et al. (1–3) recently reported that depressive symptoms themselves can predict new-onset dual sensory impairment, suggesting a reciprocal pathway that warrants further investigation using cross-lagged panel models.

Fourth, despite harmonization, cultural differences in reporting mental health symptoms (e.g., somatization in Asian cohorts) may still influence the observed geopolitical heterogeneity.

Additionally, substantial between-cohort heterogeneity was observed, partly reflecting differences in sensory impairment definitions and cohort-specific sample distributions, particularly the unusually high DSI prevalence in CHARLS and the very small DSI sample in MHAS. Finally, because sensory status was assessed under usual conditions, including possible use of corrective devices, self-reported impairment may partly reflect disparities in healthcare access, assistive device use, and treatment adequacy rather than biological decline alone. Sensory function was measured only at baseline, and changes during follow-up were not captured, which may underestimate the psychological impact of incident or progressive impairment over time. Furthermore, some potentially important confounders, including objective social isolation and baseline cognitive function, were not harmonized across all cohorts and therefore could not be fully adjusted for in the pooled analysis.

### 4.7 Conclusion

In conclusion, this global longitudinal study demonstrates that sensory impairment, particularly dual impairment, is robustly and universally associated with incident depression among older adults. The association is graded, cumulative, and culturally nuanced, with significant implications for health equity. Addressing sensory health is a critical, yet often overlooked, component of preventing late-life depression.

## Data Availability

Publicly available datasets were analyzed in this study. This data can be found at: The data will be available to other researchers for non-commercial purposes upon request to the corresponding author. HRS: https://hrs.isr.umich.edu, ELSA: https://www.elsa-project.ac.uk, SHARE: https://share-eric.eu, CHARLS: https://charls.pku.edu.cn, KLoSA: https://survey.keis.or.kr/eng/klosa/klosa01.jsp, and MHAS: https://www.mhasweb.org.

## Footnotes

1
https://www.who.int/news-room/fact-sheets/detail/ageing-and-health

2
https://www.who.int/news-room/fact-sheets/detail/mental-health-of-older-adults

3
https://www.who.int/news-room/fact-sheets/detail/blindness-and-visual-impairment

4
https://www.who.int/news-room/fact-sheets/detail/deafness-and-hearing-loss

5
https://www.who.int/teams/maternal-newborn-child-adolescent-health-and-ageing/ageing-and-health/integrated-care-for-older-people-icope
