# Supplementary material for: Sensory impairment and risk of incident depression in older adults: a harmonized longitudinal analysis of 72,177 individuals across six international cohorts
Source: Front Public Health. 2026 Jul 8;14:1834248. doi: 10.3389/fpubh.2026.1834248 (PMC13388928; doi:10.3389/fpubh.2026.1834248)
Supplement: Supplementary file 1 [file Data_Sheet_1.docx]

### Appendix A Description of Baseline and Follow-up Waves across 6 Harmonized Longitudinal Cohorts (2010–2020)

| Cohort | Country/Region | Baseline (T0) | Survey Year (T0) | Follow-up Waves Included | End of Follow-up | Total Waves Used |
| --- | --- | --- | --- | --- | --- | --- |
| HRS | USA | Wave 10 | 2010 | W11, W12, W13, W14, W15 | 2020 | 6 |
| ELSA | England | Wave 5 | 2010-2011 | W6, W7, W8, W9 | 2018-2019 | 5 |
| SHARE | Europe | Wave 4 | 2011 | W5, W6, W7*, W8 | 2019-2020 | 5 |
| CHARLS | China | Wave 1 | 2011-2012 | W2, W3, W4, W5 | 2020** | 5 |
| KLOSA | South Korea | Wave 3 | 2010 | W4, W5, W6, W7, W8 | 2020 | 6 |
| MHAS | Mexico | Wave 4 | 2015 | W5 | 2018 | 2 |

**Notes:** Time Synchronization: All cohorts were synchronized to a baseline window of 2010–2012, with the exception of MHAS. Follow-up: Participants were followed until the latest available wave (2018–2020), ensuring a follow-up duration of approximately 8–10 years (except for MHAS). SHARE: Wave 3 (SHARELIFE) was excluded due to its retrospective nature. *Wave 7 was included as it contains standard health measures alongside life history modules. CHARLS: Wave 5 (2020) was included to maximize the follow-up duration. MHAS: Wave 3 (2012) was excluded due to extensive missing data on sensory impairments; thus, Wave 4 (2015) was selected as the baseline, resulting in a shorter follow-up period (3 years).

### Appendix B Step-by-step derivation of the final analytical sample across six harmonized longitudinal cohorts

| **Exclusion Step** | **CHARLS** | **HRS** | **ELSA** | **SHARE** | **KLOSA** | **MHAS** |
| --- | --- | --- | --- | --- | --- | --- |
| Total observations in harmonized files | **96628** | **115116** | **47722** | **259718** | **44273** | **31893** |
| Excluded: Invalid follow-up duration (≤ 0) or missing | -25623 | -10638 | -4162 | -51676 | -11783 | -9795 |
| Excluded: Non-baseline waves | -59911 | -85597 | -34831 | -174630 | -27350 | -13630 |
| Excluded: Prevalent depression at baseline or missing | -157 | -2174 | -662 | -725 | -40 | -0 |
| Excluded: Age < 50 years or missing | -2691 | -793 | -134 | -694 | -216 | -309 |
| **Excluded: Missing key covariates** |  |  |  |  |  |  |
| Sensory status (Exposure) | -919 | -43 | -22 | -78 | -0 | -567 |
| Sex | -0 | -0 | -0 | -0 | -0 | -0 |
| Education | -1 | -4 | -672 | -0 | -0 | -78 |
| Marital status | -0 | -3 | -1 | -498 | -0 | -0 |
| Employment status | -120 | -148 | -1 | -168 | -0 | -14 |
| Smoking status | -5 | -99 | -196 | -6 | -0 | -4 |
| Household wealth | -1146 | -0 | -142 | -0 | -28 | -0 |
| Chronic conditions | -0 | -0 | -0 | -0 | -0 | -0 |
| Final Analytical Sample | **6055** | **15617** | **6910** | **31243** | **4856** | **7496** |

**Note:** Data are presented as the number of observations. The selection process applied a sequential exclusion strategy to ensure data quality and comparability across cohorts. Initial merged observations: Represents the total number of observations available in the harmonized data files prior to wave selection. Exclusion of non-baseline waves: We restricted the sample to the designated baseline wave for each cohort to synchronize the study period: Wave 10 (2010) for HRS, Wave 5 (2010) for ELSA, Wave 4 (2011) for SHARE, Wave 1 (2011) for CHARLS, Wave 3 (2010) for KLoSA, and Wave 4 (2015) for MHAS. Exclusion of baseline depression: Participants with prevalent depression (CES-D score ≥ cutoff) at baseline were excluded to establish a cohort at risk for incident depression. Missing data: Participants with item non-response on sensory status (vision/hearing), sociodemographic covariates (age, sex, education, marital status, wealth, employment), or health indicators (smoking, chronic conditions) were excluded to facilitate complete-case analysis. Abbreviations: CHARLS=China Health and Retirement Longitudinal Study; HRS=Health and Retirement Study; ELSA=English Longitudinal Study of Ageing; SHARE=Survey of Health, Ageing and Retirement in Europe; KLoSA=Korean Longitudinal Study of Ageing; MHAS=Mexican Health and Aging Study.

### Appendix C Stratified associations between baseline sensory impairment and incident depression across sociodemographic subgroups in the pooled dataset

| **Subgroups** | **Vision Impairment Only** | **Hearing Impairment Only** | **Dual Sensory Impairment** |
| --- | --- | --- | --- |
| **Age**(years) |  |  |  |
| ≥65 | 1.24  （1.172-1.312） | 1.26  （1.188-1.338） | 1.49  （1.392-1.593） |
| <65 | 1.16  （1.103-1.217） | 1.26  （1.172-1.347） | 1.37  （1.283-1.471） |
| **Employment status** |  |  |  |
| Yes | 1.18  （1.104-1.250） | 1.31  （1.204-1.416） | 1.37  （1.26-1.48） |
| No | 1.22  （1.162-1.275） | 1.26  （1.198-1.334） | 1.48  （1.397-1.569） |
| **Smoking status** |  |  |  |
| Current Smoker | 1.19  （1.095-1.285） | 1.20  （1.072-1.341） | 1.43  （1.287-1.583） |
| Non-smoker | 1.20  （1.153-1.253） | 1.29  （1.233-1.360） | 1.45  （1.372-1.526） |
| **Sex** |  |  |  |
| Male | 1.22  （1.145-1.292） | 1.26  （1.182-1.352） | 1.45  （1.352-1.557） |
| Female | 1.19  （1.137-1.249） | 1.31  （1.237-1.397） | 1.44  （1.352-1.537） |
| **Marital status** |  |  |  |
| Married / Partnered | 1.19  （1.138-1.243） | 1.28  （1.212-1.350） | 1.42  （1.343-1.503） |
| Other | 1.25  （1.171-1.339） | 1.29  （1.189-1.400） | 1.53  （1.404-1.675） |
| **Household Wealth** |  |  |  |
| Low (Tertile 1) | 1.18  （1.14-1.248） | 1.26  （1.167-1.353） | 1.42  （1.330-1.521） |
| Middle (Tertile 2) | 1.21  （1.136-1.293） | 1.32  （1.222-1.425） | 1.42  （1.303-1.546） |
| High (Tertile 3) | 1.21  （1.124-1.304） | 1.27  （1.170-1.381） | 1.51  （1.357-1.691） |
| **Educational attainment** |  |  |  |
| Less than High School | 1.17  （1.108-1.229） | 1.23  （1.151-1.306） | 1.35  （1.272-1.434） |
| High School / Vocational | 1.21  （1.138-1.291） | 1.33  （1.238-1.438） | 1.58  （1.440-1.726） |
| College or Above (High) | 1.24  （1.120-1.376） | 1.35  （1.200-1.525） | 1.678  （1.429-1.969） |

**Note:** Data are presented as Hazard Ratios (HR) and 95% Confidence Intervals (CI) derived from multivariable Cox proportional hazards models. Adjustments: All models were adjusted for age, sex, marital status, education, household wealth, employment status, smoking status, chronic conditions, and country (as a stratification variable), except where the variable was the stratifying factor (e.g., the model stratified by sex was adjusted for all covariates except sex). Abbreviations: CHARLS, China Health and Retirement Longitudinal Study; HRS, Health and Retirement Study; ELSA, English Longitudinal Study of Ageing; SHARE, Survey of Health, Ageing and Retirement in Europe; KLoSA, Korean Longitudinal Study of Ageing; MHAS, Mexican Health and Aging Study. Reference Group: The reference group for all HR calculations is "No Sensory Impairment" within each specific subgroup.

### Appendix D Forest plots of sensitivity analysis excluding incident depression events within the first 2 years of follow-up


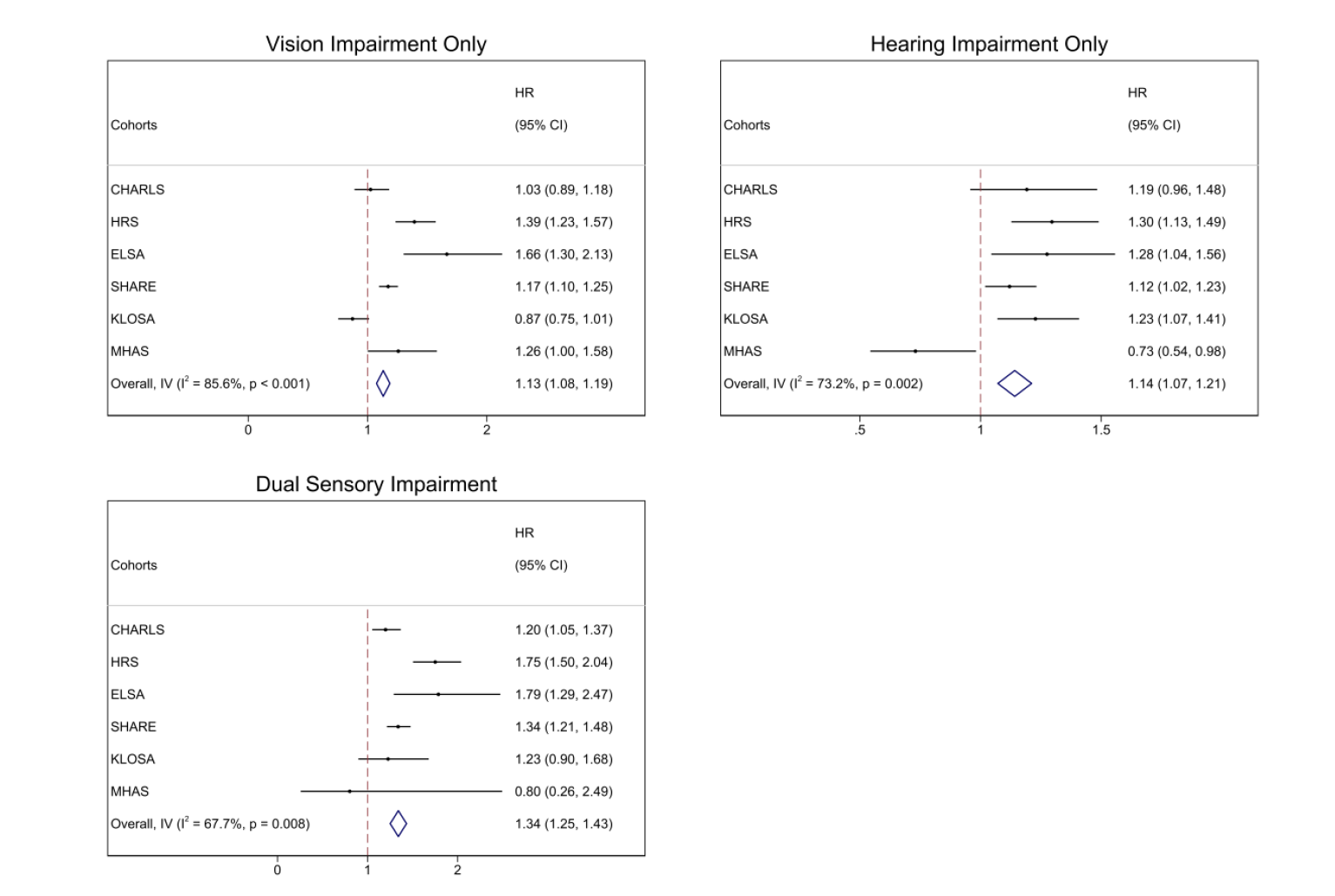


**Note:** The analysis excludes participants who developed depression or were censored within the first 2 years to minimize reverse causality. Plots display cohort-specific and pooled Hazard Ratios (HRs) with 95% Confidence Intervals (CIs) for Vision Impairment Only (top left), Hearing Impairment Only (top right), and Dual Sensory Impairment (bottom left), relative to no sensory impairment. Weights are derived from random-effects meta-analysis. Abbreviations: HR=Hazard Ratio; CI=Confidence Interval; CHARLS=China Health and Retirement Longitudinal Study; HRS=Health and Retirement Study; ELSA=English Longitudinal Study of Ageing; SHARE=Survey of Health, Ageing and Retirement in Europe; KLoSA=Korean Longitudinal Study of Ageing; MHAS=Mexican Health and Aging Study.

**Appendix E Forest plots of sensitivity analysis using logistic regression models to estimate Odds Ratios (ORs)**


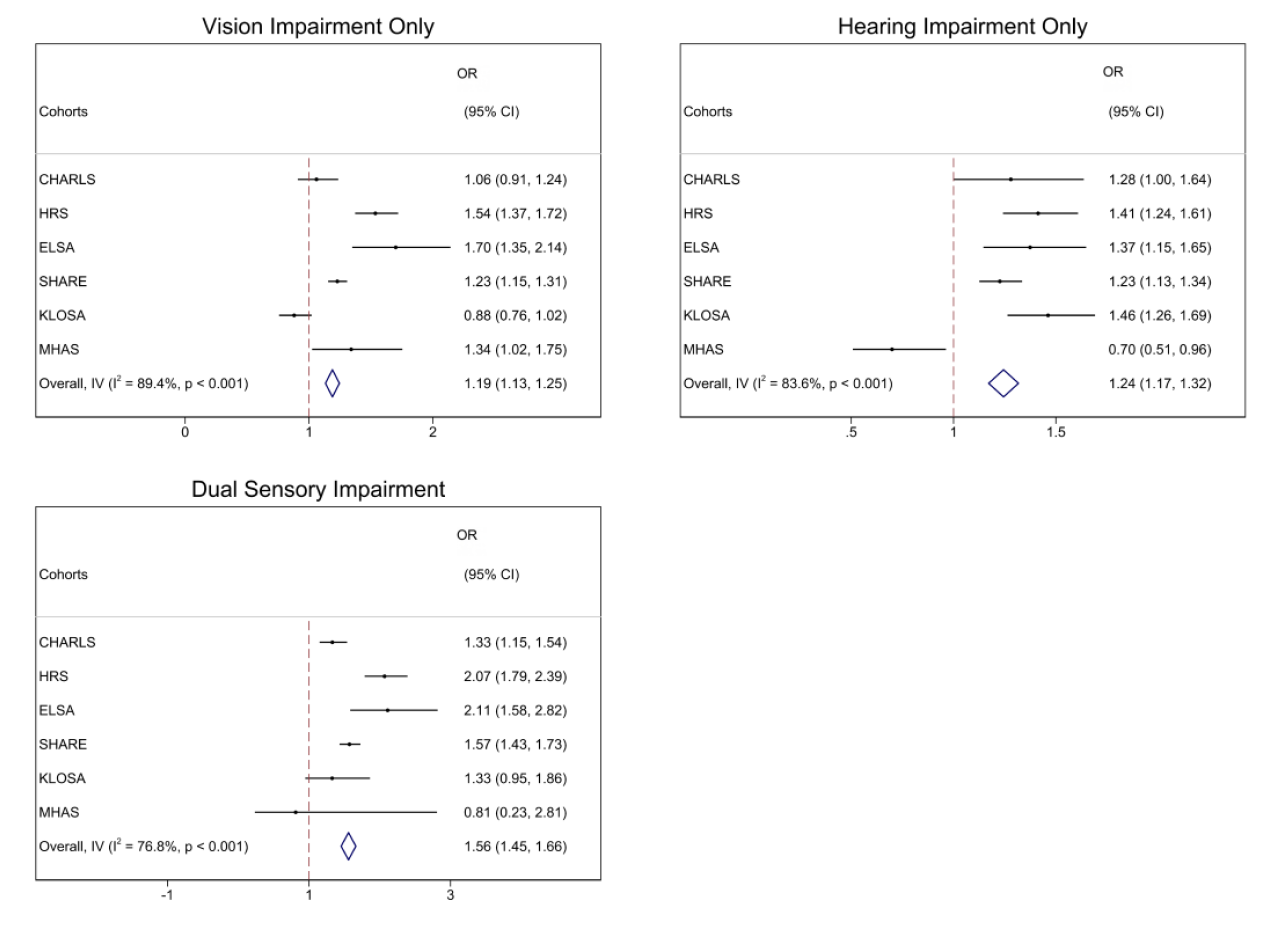


**Note:** The analysis employs multivariable logistic regression to estimate the risk of incident depression, independent of survival time. Plots display cohort-specific and pooled Odds Ratios (ORs) with 95% Confidence Intervals (CIs) for Vision Impairment Only (top left), Hearing Impairment Only (top right), and Dual Sensory Impairment (bottom left). The diamond represents the overall pooled estimate derived from random-effects meta-analysis. Abbreviations: OR=Odds Ratio; CI=Confidence Interval; CHARLS=China Health and Retirement Longitudinal Study; HRS=Health and Retirement Study; ELSA=English Longitudinal Study of Ageing; SHARE=Survey of Health, Ageing and Retirement in Europe; KLoSA=Korean Longitudinal Study of Ageing; MHAS=Mexican Health and Aging Study.
